# Aspirin enhances the sensitivity of colon cancer cells to cisplatin by abrogating the binding of NF-κB to the COX-2 promoter

**DOI:** 10.18632/aging.102644

**Published:** 2020-01-06

**Authors:** Wei Jiang, Yue Yan, Manyu Chen, Guangyu Luo, Jiaojiao Hao, Jinjin Pan, Sheng Hu, Ping Guo, Wenyang Li, Ruozu Wang, Yan Zuo, Yao Sun, Silei Sui, Wendan Yu, Zhe Pan, Kun Zou, Zongheng Zheng, Wuguo Deng, Xiaojun Wu, Wei Guo

**Affiliations:** 1 Institute of Cancer Stem Cells and The First Affiliated Hospital, Dalian Medical University, Dalian, China; 2 Sun Yat-Sen University Cancer Center, State Key Laboratory of Oncology in South China, Collaborative Innovation Center of Cancer Medicine, Guangzhou, China; 3 The Third Affiliated Hospital, Sun Yat-Sen University, Guangzhou, China

**Keywords:** aspirin, cisplatin, colon cancer, NF-κB, COX-2

## Abstract

Cisplatin is one of the most potent chemotherapeutic agents for the treatment of colon cancer. Nevertheless, the unavoidability of the notable toxicity and the development of the acquired resistance severely restricted its clinical application. Aspirin and some other non-steroidal anti-inflammatory drugs have been used to prevent colon tumorigenesis as chemopreventive agents. Here, we explored the possibility of aspirin as an adjuvant drug to boost the anti-cancer effect of cisplatin for colon cancer. We found that aspirin significantly enhanced the cisplatin-mediated inhibitions of cell proliferation, migration and invasion and the induction of apoptosis in colon cancer cells. The combined treatment of aspirin and cisplatin suppressed the expression of the anti-apoptotic protein Bcl-2 and the EMT-related proteins, up-regulated the levels of the cleaved PARP and Bax, and blocked the PI3K/AKT and RAF-MEK-ERK signaling pathway. In addition, we demonstrated that the enhanced effect of aspirin on the cisplatin-induced inhibition of tumor cell growth was also mediated through the suppression of the binding activity of NF-κB to the COX-2 promoter. The combination of aspirin and cisplatin effectively attenuated the translocation of NF-κB p65/p50 from the cytoplasm to the nucleus, and abrogated the binding of NF-κB p65/p50 to the COX-2 promoter, thereby down-regulating COX-2 expression and PGE_2_ synthesis. Moreover, the *in vivo* study also verified the enhanced anti-tumor activity of such combined therapy in colon cancer by targeting the NF-κB/COX-2 signaling. Our results provided new insights into understanding the molecular mechanisms of aspirin in sensitizing cisplatin-mediated chemotherapeutic effect in colon cancer and indicated a great potential of this combined therapy for cancer treatment.

## INTRODUCTION

Colorectal cancer is the third common cancer and the fourth frequent cause of cancer death globally, with a rough estimate of 1.2 million new cases and 600,000 deaths per year. The morbidity of colonic malignancy tends to increase with the age. Although colon cancer is more common in developed countries, the colon risk was found to be increased rapidly in developing countries [1–3]. The surgical excision is effective for over 80% of colorectal carcinomas, nevertheless, 50% of patients subsequently relapse with metastasis. Hence, adjuvant treatment soon after surgery is especially necessary. Continuous radiochemotherapy treatment was widely taken in clinic to eliminate residual tumor cells, prolong disease-free intervals and improve overall survival [4]. However, the gradually developed radiochemotherapeutic resistance and the inevitable toxicity to normal cells strictly limited their effects.

Cisplatin is a platinum-containing coordination complex used in cancer treatment [5, 6]. It is also one of the most common chemotherapeutic agents used for the treatment of colon cancer other than 5-Fluorouracil. Accumulating evidence suggests that multiple mechanisms underlie the anticancer potential of Cisplatin, including DNA synthesis inhibition, the generation of DNA lesions and the induction of mitochondrial apoptosis via forming DNA adducts with platinum atoms [7]. However, the widespread resistance to Cisplatin renders malignant cells less susceptible to the antiproliferative and cytotoxic effects of the drug. A large fraction of Cisplatin-treated patients is destined to experience therapeutic failure and tumor recurrence. Coupled with notable adverse effects, its clinical application is limited seriously [8]. Thus, more and more researchers pay attentions to the trials of adjuvant drugs which can be combined with Cisplatin to exert a more significant antineoplastic effect and minimize the toxic and side effects of Cisplatin.

Like other non-steroidal anti-inflammatory drugs (NSAIDs), Aspirin has been shown to functionalize as the potentially effective chemopreventive agent for colorectal cancer [9–11]. Moreover, an adjuvant chemotherapy trial carried out by Chan et al. in 1279 patients with stage I-III colorectal cancer showed that use of Aspirin after diagnosis of nonmetastatic colorectal cancer was associated with improved survival from the disease, particularly among individuals with primary tumors that overexpress COX-2 [12]. Multiple molecular mechanisms seem to be involved in the tumor suppressive effect of Aspirin. Accumulating evidence indicates that Aspirin targets the COX-2 enzyme to suppress the production of potentially neoplastic prostaglandins arising from COX-2-mediated catalysis of arachidonic acid [13, 14]. Some present studies provide evidence that Aspirin inhibits the growth of colorectal cancer stem cells by disrupting the Wnt/β-catenin signal pathway [15]. In addition, Aspirin has been reported to dysregulate NF-κB signaling and insulin-like growth factor I. It has been demonstrated that Aspirin could bind to and inactive IκB kinase-β (IKK-β), which in turn prevents the degradation of IκB and the subsequent translocation of NF-κB to the nucleus, where it activates the transcription of a variety of target genes [16]. Given that dysregulation of the NF-κB signaling pathway is a common event in colon cancer, which contributes to tumor initiation and progression by driving expression of pro-proliferative/ anti-apoptotic genes, the inactivation of NF-κB is intended as a new strategy to eliminate colon cancer cells through induction of apoptosis [17]. Thus, we raised the important question of whether Aspirin combined with Cisplatin could elevate the anti-tumor effect of Cisplatin and alleviate its side effects in colon cancer treatment.

Here, the combined application of non-steroidal anti-inflammatory drug Aspirin and traditional chemotherapeutant Cisplatin in colorectal cancer treatment has been advocated. We found that Aspirin combined with Cisplatin led to more significant cancer cell proliferation, migration and invasion inhibition and apoptosis induction in vitro accompanied by the simultaneous inactivation of the PI3K/AKT and RAF-MEK-ERK signaling pathway. Intriguingly, such combined treatment antagonized tumor growth partially by suppressing COX-2 expression and PGE_2_ production via a mechanism dependent of dysregulated NF-κB signaling. Furthermore, the synergistic anti-tumor efficiencies benefit from the combinational usage of these two drugs and the underlying molecular mechanisms were also evaluated and confirmed in athymic nude mice with xenografts of human colon cancer cells. All the findings revealed a role for Aspirin in sensitizing colon cancer cells to Cisplatin treatment and provided a basis for the potential of their combined application in clinical setting for colon cancer therapy.

## RESULTS

### Aspirin potentiated the cisplatin-mediated inhibition on cell viability

**Figure 1:**
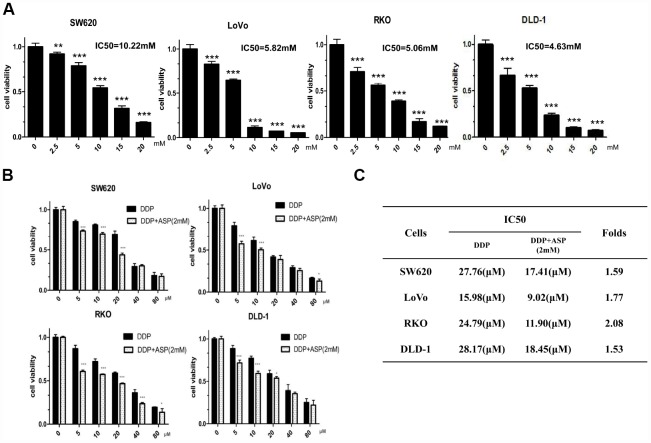
**Aspirin synergizes the inhibiting effect of Cisplatin on cell viability in colon cancer cells.** (**A**) Human colon cancer cells SW620, LoVo, RKO and DLD-1 were treated with different concentrations of Aspirin for 48 h, and the cell viability was tested by MTT assay (n=6). (**B**) Human colon cancer cells were treated with indicated dose of Cisplatin alone or Cisplatin combined with 2 mM Aspirin for 48 h, and the cell viability was tested by MTT assay (n=6). (**C**) IC50 values of Cisplatin were calculated by software CVXPT32 in colon cancer cells upon treatment with Cisplatin alone or Cisplatin combined with 2 mM Aspirin. Data were presented as means ± SD, *P<0.05, **P<0.01, ***P<0.001.

MTT assay was performed to measure IC50 values of aspirin in different colon cancer cells with drug treatment for 48 hours. Aspirin significantly inhibited cell viabilities in a dose-dependent manner in colon cancer SW620, LoVo, RKO and DLD-1 cells (Figure 1A). Then, the synergistic inhibiting effect on cell viability under co-treatment of Aspirin and Cisplatin was evaluated. As shown in Figure 1B, the combination of Aspirin (2mM) and Cisplatin (5μM, 10μM, 20μM, 40μM, 80μM) decreased colon cancer cell viabilities more efficiently. In addition, the combined treatment resulted in a remarkable reduction of IC50 values for Cisplatin (Figure 1C), demonstrating that Aspirin improved Cisplatin-mediated cell viability inhibition in colon cancer cells.

### Aspirin and Cisplatin synergistically inhibited cell proliferation and changed cell morphology

**Figure 2:**
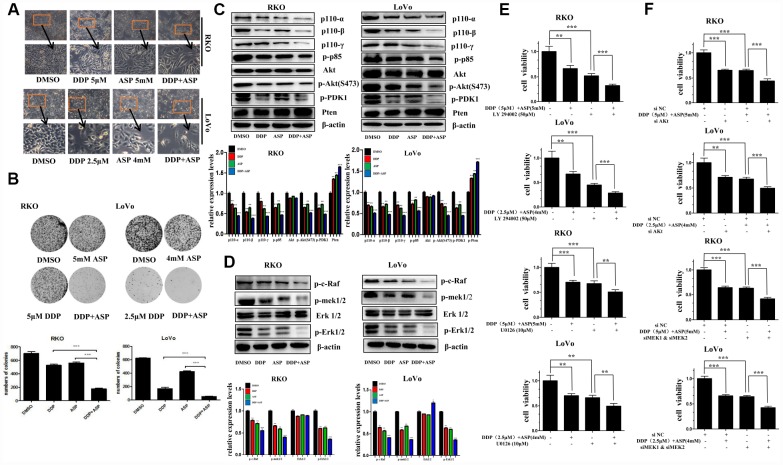
**Aspirin synergizes the inhibiting effect of Cisplatin on cell proliferation in colon cancer cells.** (**A**) Human colon cancer cells RKO and LoVo were treated with Cisplatin alone or Aspirin alone or their combination at indicated dose for 48 h. The representative images of cell morphology were taken by inverted microscope, Scale bars, 200 μm. (**B**) The colony formation assay of human colon cancer cells RKO and LoVo treated with Cisplatin alone or Aspirin alone or their combination at indicated dose was taken to detect the capacity of cell proliferation (n=4). (**C**) The expression levels of main PI3K/AKT signaling pathway-related proteins p110-α, p110-β, p110-γ, p-p85, p-PDK1, Akt, p-Akt(S473) and Pten in human colon cancer cell RKO and LoVo treated with Cisplatin alone (15 μM) or Aspirin alone (10 mM) or their combination for 48 h were detected by Western blot assay (n=3). (**D**) Human colon cancer cells RKO and LoVo were treated with Cisplatin alone (15 μM) or Aspirin alone (10 mM) or their combination for 48 h, and the expression levels of main RAF-MEK-ERK signaling pathway-related proteins p-c-Raf, p-mek1/2, Erk1/2 and p-Erk1/2 were detected by western blot assay (n=3). (**E**) Human colon cancer cells RKO and LoVo were pretreated with PI3K inhibitor LY294002 (50 μM) or MEK inhibitor U0126 (10 μM) for 8 h, and then incubated with combination of Aspirin and Cisplatin. After 48 h, cell viability was determined by MTT analysis (n=6). (**F**) Human colon cancer cells RKO and LoVo were transfected with specified siRNAs for 24 hours, and then incubated with the combination of Aspirin and Cisplatin. After 48 h, cell viability was determined by MTT analysis (n=6). All the data were presented as means ± SD, *P<0.05, **P<0.01, ***P<0.001.

Cell malignant proliferation plays a pivotal role in colon carcinogenesis. Firstly, we detected the effect of the combined treatment on cell morphological changes. Compared with the cells treated with Aspirin alone or Cisplatin alone, the cells co-treated with Aspirin and Cisplatin started to become swollen and flat, with fewer formation of filopodia and lower spreading (Figure 2A). Then, colony formation assay was employed to evaluate the influence of the combined treatment on the clonogenic capacities. A remarkable decreased colony formation ratio was seen in cells treated with the combination of Aspirin and Cisplatin (Figure 2B).

Since the aberrant activation of PI3K-Akt signaling and RAF-MEK-ERK signaling pathway has been reported to be involved in colon cancer proliferation [19–21], we next detected the expression levels of some key proteins involved in PI3K-Akt and RAF-MEK-ERK signaling pathway by western blot assay in colon cancer cells upon co-treatment with Aspirin and Cisplatin. As shown in Figure 2C and 2D, compared to single drug treatment group, the expression of the PI3K/AKT signaling pathway related proteins p110-α, p110-β, p110-γ, p-p85, p-PDK1, p-Akt(S473), and the RAF-MEK-ERK signaling pathway related proteins p-c-Raf, p-mek1/2, p-Erk1/2 were all suppressed more significantly by the combined treatment of Aspirin and Cisplatin. Consistently, the combined treatment caused more increase of Pten expression level, the important negative regulator of PI3K-Akt signaling pathway, but almost had no effect on the expression of total Akt and Erk1/2.

To further confirm the involvement of PI3K-Akt and RAF-MEK-ERK signaling pathways in the synergistic anti-tumor efficacy caused by the combined usage of Aspirin and Cisplatin, LY294002, a widely used pan-PI3K inhibitor [22], and U0126, a potent small molecule MEK1/2-selective inhibitor [23] was respectively used to combine with Aspirin and Cisplatin to co-treat colon cancer cells. Such combined treatment markedly enhanced the inhibitory rate on cell viability in cancer cells, by comparison to Aspirin and Cisplatin co-treatment (Figure 2E). When Akt or MEK1/2 was knocked down using their specific siRNAs to inhibit relevant signaling pathways, the consistent results were obtained (Figure 2F). Given the results above, all these findings indicated that the synergistic inhibition on cell proliferation caused by the combined treatment of Aspirin and Cisplatin was at least partially mediated via PI3K-Akt and RAF-MEK-ERK signaling pathway.

### Aspirin and Cisplatin synergistically inhibited cell migration and invasion

**Figure 3:**
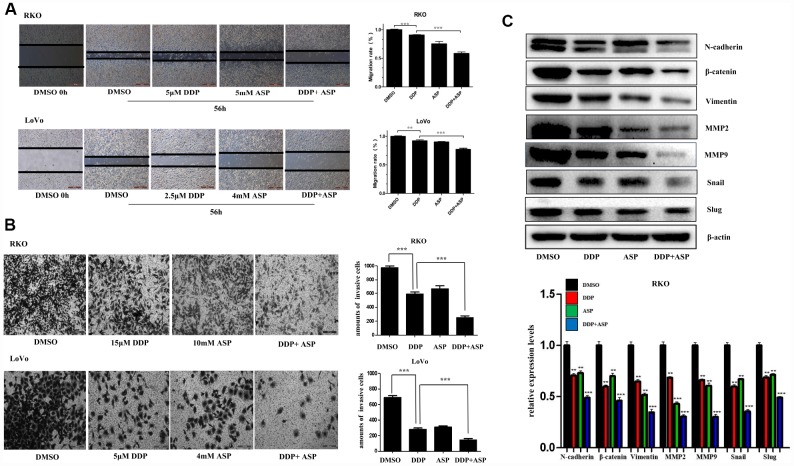
**Aspirin increased the inhibiting effect of Cisplatin on cell migration and invasion in colon cancer cells.** (**A**) A wound-healing assay of human colon cancer cells RKO and LoVo treated with Cisplatin alone or Aspirin alone or their combination at indicated dose for 56 h was performed to detect the capacity of cell migration. Scale bars, 500 μm. The cell migration rate was calculated through the quantification of migration distance (n=4). (**B**) The capacity of cell invasion of human colon cancer cells RKO and LoVo treated with Cisplatin alone or Aspirin alone or their combination at indicated dose for 96 h was analyzed by a transwell assay. The invaded cells were stained by crystal violet and the representative images were taken by inverted microscope (n=4). Scale bars, 200 μm. (**C**) The expression levels of main EMT- related proteins N-cadherin, β-catenin, Vimentin, MMP2, MMP9, Snail and Slug in human colon cancer cell RKO treated with Cisplatin alone (15 μM) or Aspirin alone (10 mM) or their combination for 48 h were detected by western blot assay (n=3). Data were presented as means ± SD, *P<0.05, **P<0.01, ***P<0.001.

Epithelial-to-mesenchymal transition (EMT), which appears to be closely involved in the pathogenesis of colon cancer, plays a critical role in driving colon cancer invasion and metastasis. During EMT procedure, cell migratory and invasive properties would be enhanced considerably [24, 25]. We then evaluated the effects of the combined usage of Aspirin and Cisplatin on cell migration and invasion. The results of scratch assay and transwell assay, shown in Figure 3A and 3B, revealed that the combined treatment of Aspirin and Cisplatin imposed a more powerful inhibiting effect on colon cancer cell migration and invasion. Consistently, the combined treatment of Aspirin and Cisplatin obviously attenuated the expression of main EMT-related proteins N-cadherin, β-catenin, Vimentin, MMP2, MMP9, Snail and Slug in human colon cancer cells (Figure 3C).

### Aspirin increased the cisplatin-induced apoptosis by activating cytochrome-c/PARP signaling

**Figure 4:**
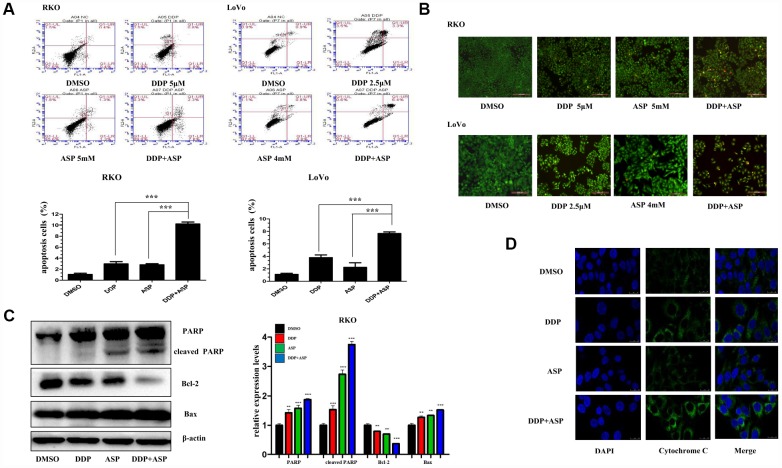
**Aspirin enhanced the Cisplatin-induced apoptosis in colon cancer cells.** (**A**) A FACS analysis was used to detect cell apoptosis in human colon cancer cells RKO and LoVo treated with Cisplatin alone or Aspirin alone or their combination at indicated dose for 48 h. The percentage of apoptotic cells was further calculated (n=4). (**B**) Human colon cancer cells RKO and LoVo treated with cisplatin alone or aspirin alone or their combination at indicated dose for 48 h were stained with acridine orange and ethidium bromide to observe cell apoptosis. The representative images were taken by inverted fluorescence microscope. Scale bars, 200 μm (n=3). (**C**) The expression levels of main apoptosis-related proteins PARP, cleaved PARP, Bcl-2 and Bax in human colon cancer cell RKO treated with Cisplatin alone (15 μM) or Aspirin alone (10 mM) or their combination for 48h were detected by western blot assay (n=3). (**D**) Immunofluorescence assay of human colon cancer cell LoVo treated with Cisplatin alone (2.5 μM) or Aspirin alone (4 mM) or their combination for 48 h was implemented to monitor cytochrome-c release from intermembrance space of mitochondria into cytoplasm. The representative images were taken by laser scanning confocal microscope. Scale bars, 25 μm (n=3). Data were presented as means ± SD, *P<0.05, **P<0.01, ***P<0.001.

Evidences are increasingly available to support the hypothesis that the evasion of apoptosis is one of the prominent hallmarks in the evolution of colon cancer and leads to cellular poor response to chemotherapy and radiotherapy [26, 27]. Hence, we further investigated the influence of Aspirin on Cisplatin-induced apoptosis. By using Annexin V-FITC/PI staining-based FACS analysis, we found that the addition of Aspirin greatly increased the Cisplatin-induced apoptosis in colon cancer cells (Figure 4A). To detect the apoptotic morphological changes of the colon cancer cells treated with Aspirin and Cisplatin, AO/EB staining assay was performed. Consistent with FACS analysis, more early apoptotic cells (yellow), late stage apoptotic cells (orange) and even dead cells (red) were seen in the combined treatment group. Nuclear condensation and apoptotic bodies formation were also clearly observed in the group treated with the combined compounds (Figure 4B). Moreover, the combined treatment notably improved the expression levels of PARP, cleaved PARP and Bax, down-regulated the expression of Bcl-2 (Figure 4C) and triggered more cytochrome-c release from the inter-mitochondrial space to cytosol (Figure 4D). Together, these results demonstrated that Aspirin and Cisplatin synergistically enhanced the induction of cell apoptosis through activating intrinsic mitochondrial pathway.

### Aspirin and Cisplatin synergistically down-regulated the expression of COX-2 by inhibiting NF-κB signaling pathway

Constitutive activation of NF-κB is frequently observed in colon cancer, and plays a key role in antiapoptosis, promoting tumor growth and drug resistance [28, 29]. Under the cellular stimulation by numbers of inflammatory factors, IκB is phosphorylated by the IκB kinase (IKK) complex, and then degraded by the 26S proteasome. Subsequently, the heterodimer of the p50 and p65 translocates to the nucleus, where it binds to the promoter region of target genes, including c-myc, p53 and COX-2 [30].

**Figure 5:**
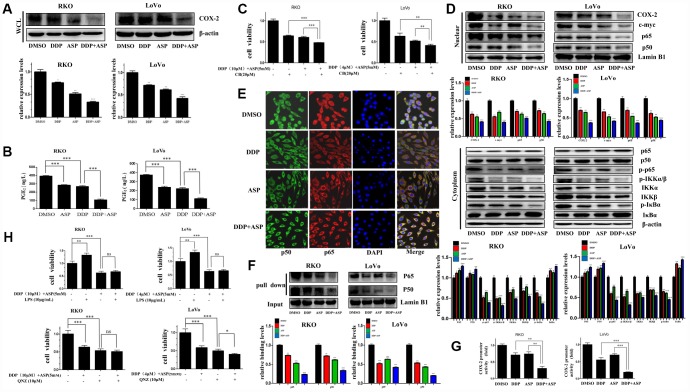
**Cotreatment with Aspirin and Cisplatin suppressed NF-κB/COX-2 signaling pathway in colon cancer cells.** (**A**) The expression level of COX-2 in the whole cell lysate of human colon cancer cells RKO and LoVo treated with Cisplatin (15μM /5μM) and/or Aspirin(10mM /5mM) for 48 hours was analyzed by Western blot (n=3). (**B**) Human colon cancer cells RKO and LoVo were treated with Cisplatin (15μM /5μM) and/or Aspirin (10mM /5mM) for 48 h. The amount of PGE_2_ in cell culture media was detected by Prostaglandin E_2_ High Sensitivity in vitro competitive ELISA Kit (n=4). (**C**) Human colon cancer cells RKO and LoVo were incubated with combination of Aspirin and Cisplatin at indicated dose for 48 h after pretreatment with COX-2-selective inhibitor celecoxib (CB) at indicated dose for 8 h, and then cell viability was determined by MTT analysis (n=6). (**D**) Human colon cancer cells RKO and LoVo were treated with Cisplatin alone (15μM /5μM) or Aspirin alone (10mM /5mM) or their combination for 48 h. The expression levels of p65, p50, COX-2 and c-myc in nucleus, p65, p50, p-p65, IKKα, IKKβ, p-IKKα/β, p-IκBα and IκBα in cytoplasm were respectively detected by western blot assay (n=3). (**E**) Immunofluorescence assay of human colon cancer cell RKO treated with Cisplatin alone (15μM) or Aspirin alone (10mM) or their combination for 48 h was implemented to observe the subcellular localization of p65 and p50. The representative images were taken by laser scanning confocal microscope. Scale bars, 25 μm (n=3). (**F**) The streptavidin-biotin pulldown assay was performed to test the binding of p65 and p50 at COX-2 promoter region in human colon cancer cells RKO and LoVo treated with Cisplatin (15μM /5μM) and/or Aspirin (10mM /5mM) for 48 h (n=3). (**G**) Human colon cancer cells RKO and LoVo were transfected with COX-2 promoter (-892/+9 fragments) driven-luciferase plasmids and pRL-TK Renilla luciferase construct (Promega). After 24 hours, the cells were treated with Cisplatin (15μM /5μM) and/or Aspirin (10mM /5mM) for 48 hours. Then luciferase activities were measured according to the Dual-Luciferase Assay System protocol (Promega) (n=4). (**H**) Human colon cancer cells RKO and LoVo were pretreated with NF-κB activator LPS or NF-κB inhibitor QNZ at indicated dose for 8 h, and then incubated with combination of Aspirin and Cisplatin at indicated dose. After 48 h, cell viability was determined by MTT analysis (n=6). Data were presented as means ± SD, *P<0.05, **P<0.01, ***P<0.001.

Given that cyclooxygenase-2 (COX-2) is a known downstream effector of Aspirin contributing to its anti-inflammation and tumor-suppressing effects, and the aberrant activation of COX-2/PGE_2_ signaling axis has a critical role in promoting colon carcinogenesis [31, 32], we next evaluated whether the synergistic anti-tumor efficacy caused by the combinational usage of Aspirin and Cisplatin was similarly realized by targeting COX-2. Western blot analysis indicated that Aspirin combined with Cisplatin effectively inhibited the expression of COX-2 in the whole cell lysate (Figure 5A). Consistently, their combined treatment intensely restrained PGE_2_ production in vitro based on the results of ELISA assay (Figure 5B). Further, the combined treatment with three drugs, Aspirin, Cisplatin and Celecoxib (CB), a widely known COX-2-selective inhibitor [33], distinctly enhanced the inhibiting effect on colon cancer cell viability compared to the combined treatment of Aspirin and Cisplatin (Figure 5C). These data indicated that the elevated cell growth inhibition by the combined treatment of Aspirin and Cisplatin might partially be realized through blocking the COX-2/PGE_2_ signaling pathway.

The level of COX-2 is known to be strictly regulated by NF-κB signaling [34]. Therefore, we asked whether Aspirin combined with Cisplatin inhibited COX-2 expression by inactivating NF-κB signaling. We determined the expression levels of p50, p65, c-myc and COX-2 in nucleus and found that all these proteins were reduced after the combined treatment significantly. Furthermore, a certain down-regulation of p-p65, IKKα, IKKβ, p-IKKα/β and p-IκBα expression, and the increased expression of p65, p50 and IκBα in cytoplasm were observed in the combined treatment group (Figure 5D). To further validate the influence of combined treatment on the subcellular localization of NF-κB p65/p50, the immunofluorescence assay was performed. Consistent with the above results of western blot assay, Aspirin synergized with Cisplatin markedly attenuated the translocation of NF-κB p65/p50 (Figure 5E). In addition, pull down assay indicated that the binding of p65/p50 to the COX-2 promoter was reduced dramatically after the combined treatment of Aspirin and Cisplatin (Figure 5F). Dual-luciferase reporter assay further showed that Aspirin and Cisplatin synergistically suppressed COX-2 promoter activity (Figure 5G), thereby suppressing COX-2 expression.

To further confirm the involvement of the dysregulated NF-κB signaling pathway in the synergized anti-cancer effect mediated by Aspirin and Cisplatin co-treatment, we used Lipopolysaccharides (LPS) [35, 36] or the 6-amino-4-(4-phenoxyphenylethylamino) quinazoline (QNZ) [37] to respectively activate or suppress NF-κB signaling in colon cancer cells following the co-treatment of Aspirin and Cisplatin and analyzed cell viability changes. LPS treatment couldn’t effectively rescue the restriction that the combined treatment of Aspirin and Cisplatin imposed on colon cancer cell viability. The combined treatment of QNZ with Aspirin and Cisplatin also did not substantially intensify the inhibiting effect on cell viability in RKO cells, while caused certain enhanced block in LoVo cells (Figure 5H). These data further demonstrated that the dysregulation of NF-κB signaling pathway was mainly responsible for the cell growth arrestment induced by the combined treatment of Aspirin and Cisplatin. According to all the evidence above, the down-regulation of COX-2/PGE_2_ mediated by the blockage of NF-κB signaling pathway was identified to be the main molecular mechanism by which the combined treatment of Aspirin and Cisplatin achieved better anti-tumor growth function.

### Aspirin and Cisplatin synergistically suppressed the growth of colon cancer xenografts in nude mice

**Figure 6:**
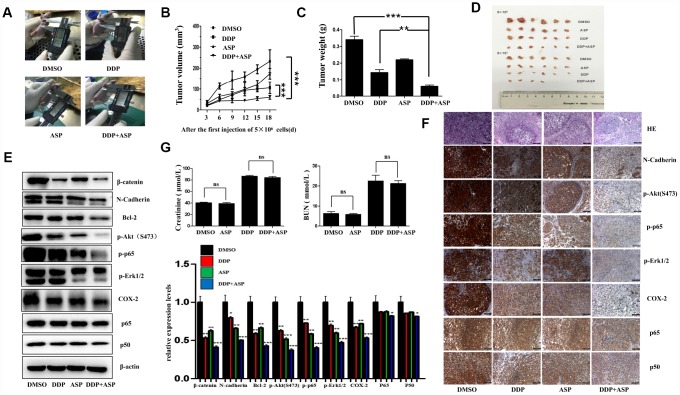
**Aspirin synergizes the inhibiting effect of Cisplatin on tumor growth in a xenograft mouse model of human colon cancer cells.** Human colon cancer LoVo cells (5×10^6^, 5×10^5^ in 100 ul PBS) were injected subcutaneously into the left and right flank of each athymic nude mice respectively. The four randomly assigned groups (n=6 for each group) were used: (1) non-drug therapy as negative control; (2) the treatment with Cisplatin (3 mg/kg) through intraperitoneal injection every three days; (3) a daily treatment of Aspirin(100 mg/kg) through intragastric administration; (4) the combination therapy of Cisplatin and Aspirin. (**A**) The representative images of the measurement of tumor diameters. (**B**) Dynamic development of tumor volume during the therapy. (**C**) Tumor weight of nude mice from each group at the moment when mice were sacrificed. (**D**) Images of xenograft tumor harvested after therapy. (**E**) The expression levels of β-catenin, N-Cadherin, Bcl-2, p-Akt(S473), p-p65, p-Erk1/2, COX-2, p65 and p50 in tumor tissue lysates were detected by western blot assay (n=6). (**F**) HE staining and immunohistochemical staining assay to show tissue morphological variations and the expressions of N-Cadherin, p-Akt(S473), p-p65, p-Erk1/2, COX-2, p65 and p50 in tissue sections. The representative images were taken by upright microscope. Scale bars, 100 μm (n=6). (**G**) Serum creatinine (Cr) and Blood Urea Nitrogen (BUN) levels of mice in each group were measured by the detection kit (n=6). Data were presented as means ± SD, *P<0.05, **P<0.01, ***P<0.001.

Based on the results of *in vitro* studies, we further explored the *in vivo* anti-cancer effect of the combined drug treatment by employing a xenograft model. Nude mice were injected subcutaneously with 5×10^6^/5×10^5^ LoVo cells into the left/right flank. When the tumors implanted on the left flanks reached 30 mm^3^, Aspirin and/or Cisplatin was administered continuously for 19 days, and the therapeutic efficiencies were evaluated. As shown in Figure 6A–6D, both the tumor volume (Figure 6A, 6B, 6D) and the tumor weights (Figure 6C) in the co-treated mice were decreased significantly. In addition, the western blot analysis of tissue lysates from xenograft tumors showed that the combined therapy markedly suppressed the expression of β-catenin, N-Cadherin, Bcl-2, p-Akt(S473), p-p65, p-Erk1/2 and COX-2 *in vivo*, while nearly caused no change of total levels of p65 and p50 (Figure 6E). Similarly, the down-regulation of the expression of these proteins in the co-treated group was confirmed by IHC assay (Figure 6F). Intriguingly, in accordance with the *in vitro* data, the results of immunostaining had born out that combined treatment blocked the nuclear translocation of NF-κB p65/p50 *in vivo*. Moreover, the H&E staining displayed that the tumor cells were irregular, deep-colored, and arranged closely with larger and abnormal nuclei and nuclear pleomorphism in the untreated group. All these results supported that the combined therapy effectively inhibited tumor growth *in vivo*, and such roles were at least partially played by regulating PI3K-Akt, RAF-MEK-ERK and NF-κB/COX-2 signaling pathways. Meanwhile, we further determined the nephrotoxicity possibly brought by the combined therapy. As shown in Figure 6G, Aspirin administration alone caused nearly no significant renal toxicity. By contrast, the single chemotherapy of Cisplatin dramatically increased the mice serum levels of creatinine (Cr) and Blood Urea Nitrogen (BUN), while the combination treatment group presented a slightly reduced elevation of serum Cr and BUN levels compared with Cisplatin treatment alone, implying the combination of Aspirin and Cisplatin produced a much more potent tumor growth inhibition effect with no obviously enhanced renal toxicity.

## DISCUSSION

Colorectal cancer, the second leading cause of death from cancer among adults, begins as a benign adenomatous polyp, which develops into advanced adenoma with high-grade dysplasia and then progresses to an invasive cancer as a result of complex genetic and epigenetic changes [27, 38]. Even though surgery has achieved important advances as the basic therapy for colon cancer, still 40 to 50 percent of patients who undergo potentially curative surgery alone ultimately relapse and die of metastatic disease [2, 39]. Hence, adjuvant therapeutics, including radiochemotherapy, targeting therapy and immunotherapy, appear to be of high significance in decreasing the recurrence and improving the survival rate [40]. However, the gradually developed resistance to the adjuvant therapeutics mentioned above in the course of treatment and the inevitable side effects to normal cells seriously limit their applications and compromise their therapeutic efficacies. The same prominent hurdle related to the use of Cisplatin in the clinical practice is posed [8, 41], although it is a highly effective chemotherapeutic drug and displays a broad spectrum of antitumor activities, especially favored in the treatment of colon cancer [42]. Here, we identified an unexpected function of Aspirin in sensitizing colon cancer cells to Cisplatin treatment. Cisplatin displayed remarkably improved anti-tumor efficiencies in vitro and in vivo upon co-treatment with Aspirin.

In recent years, Aspirin has become increasingly popular as a kind of preventive agent against CVD (cardiovascular disease) and CRC (colorectal cancer) in adults aged 50 to 59 years suffering a 10% or greater 10-year CVD risk [43]. In addition, a more pronounced protective effect is seen at substantially higher does (>14 tablets/wk) than those recommended for the prevention of CVD [44]. Furthermore, prediagnostic aspirin use was reported to reduce mortality by approximately one-half among women with lymph node-negative breast cancer [45]. Combined with the epidemiological data indicating that people who regularly take Aspirin or other NSAIDs have a 40-50% reduced risk of dying from colon cancer [30], and the earlier studies demonstrating that treatment of colon cancer cells with Aspirin resulted in a concentration-dependent decrease of cell proliferation, migration and invasion [46, 47], we deduce that combining Aspirin with traditional chemotherapeutic agents Cisplatin in colon cancer therapy might be of potential interest. In agreement with what we propose, the synergistic potential of Aspirin or Aspirin analogues with Cisplatins by their combined use or the use of Asplatin, a fusion compound of aspirin and cisplatin against cancer cells, including gastric cancer, oesophageal cancer and even colorectal cancer, have preliminarily been confirmed in the previous studies [48–50]. Nevertheless, these studies only stay at in vitro level and lack systematic clarification of the underlying molecular mechanisms. On contrary, our research not only demonstrates the largely enhanced inhibiting effects by the combined treatment of Aspirin and Cisplatin on colon cancer cell progression in vitro and in vivo, but also provided the potential molecular mechanisms responsible for such improved anti-cancer cytotoxicity.

Since the disruption of apoptosis is highly implicated in various malignancies, targeting apoptotic cell death has been the center of attraction for therapeutic intervention in all cancers. Cisplatin is known to play its cytotoxic role in anti-cancer therapy by forming platinum-DNA adducts triggering various cellular responses, including replication arrest, transcription inhibition and DNA repair, and finally leading to cell apoptosis. Consistently, Aspirin and other non-steroidal anti-inflammatory drugs also induce apoptosis in most cell types, which is considered to be an important mechanism for their anti-tumor activity and prevention of carcinogenesis. Therefore, reasonably, the elevated cytotoxicity to colon cancer cells mediated by the combined usage of Aspirin and cisplatin might also be realized via initiating more cell apoptosis. Here, without precedent, our data has confirmed this hypothesis. The combined treatment of Aspirin and cisplatin increased levels of apoptosis in colon cancer cells accompanied by the significant apoptotic morphological changes, more cytochrome-c release into cytosol and expression variations of apoptosis related proteins. Of note, all the changes at the molecular level mentioned above was completely consistent with the molecular events happening at the mitochondria-associated apoptotic pathways. We thereby conclude that Aspirin sensitized colon cancer cells to cisplatin-induced apoptosis by activating mitochondria-associated apoptosis signaling in colon cancer cells.

**Figure 7:**
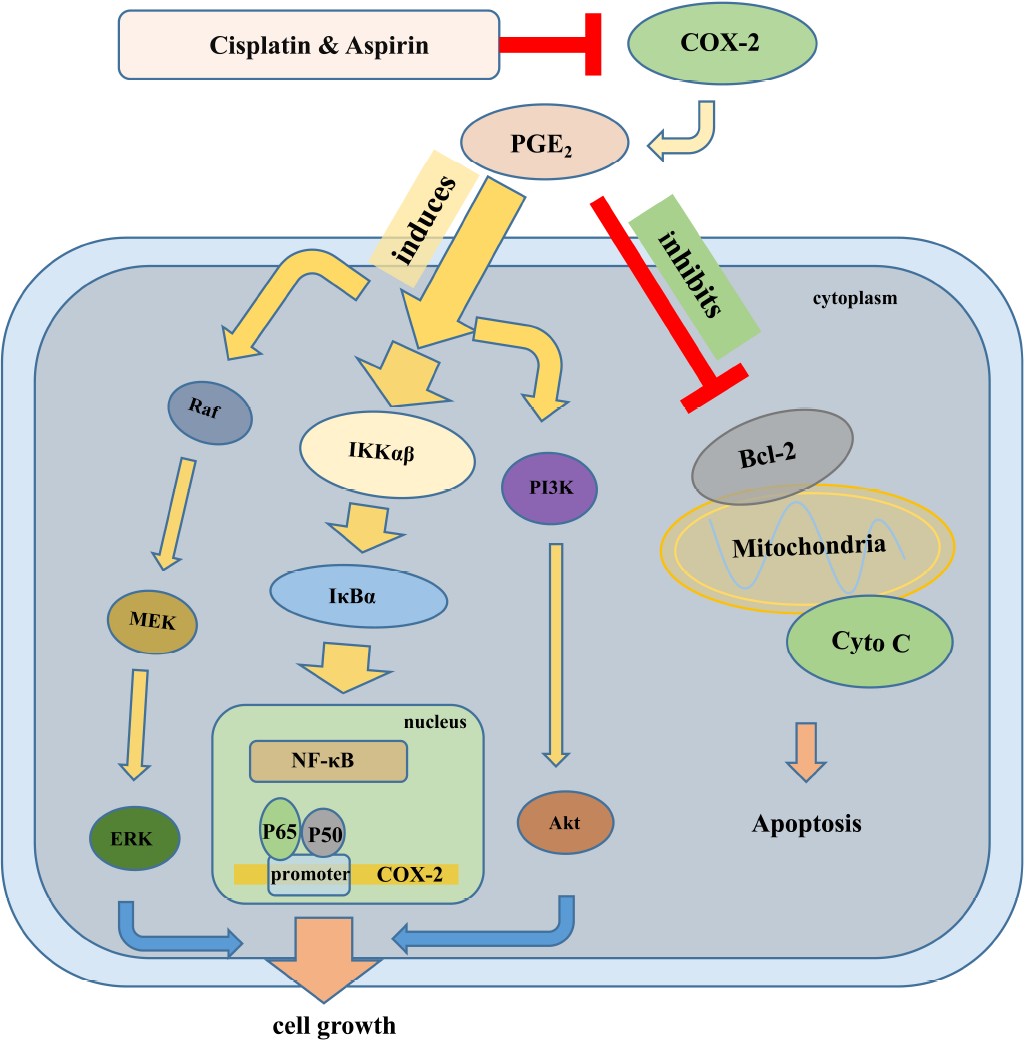
**The schematic of the molecular mechanisms involved in the synergistic anti-tumor effect of Aspirin and Cisplatin in colon cancer.** Aspirin in combination with Cisplatin enhanced the inhibition of cell proliferation, migration and invasion by reducing COX-2 mediated prostaglandin E2 synthesis and attenuating downstream PI3K/AKT, RAF-MEK-ERK and NF-κB/COX-2 signaling activities. Meanwhile, combination with Aspirin and Cisplatin could induce more apoptosis through triggering cytochrome-c release.

Our further goal for this investigation was to elucidate potential molecular mechanism of actions for the combined treatment contributing to the synergistic anticancer capacity of human colon cancer cells. Considering the specialized dysregulation of aspirin on COX-2/PGE_2_ signaling and then on PI3K/Akt, RAF/MEK/ERK, and NF-κB signaling in its anti-tumor activity [51], we hypothesized perhaps the same mechanism works in the synergistic anti-tumor effect mediated by the combined treatment. In line with our expectations, the results suggested that the significantly down-regulated PI3K/Akt, RAF/MEK/ERK, and NF-κB/COX-2/PGE_2_ signaling pathway were indeed to be responsible for the synergistic inhibition of colon cancer cell growth mediated by Aspirin combined with Cisplatin, as Aspirin combined with Cisplatin treatment caused more serious blockade of any of these three pathways compared to Aspirin treatment alone. Given that the activation of Akt inhibits GSK3 and further prevents the phosphorylation of the cytoplasmic signaling molecule β-catenin, which impedes its degradation, hence promoting its translocation to the nucleus and consequently promoting cell cycle progression [52, 53], most likely, the combined treatment of aspirin and cisplatin similarly exerts a synergistic impact on the subcellular localization of β-catenin and cell cycle distribution. Moreover, Akt also regulates the apoptotic machinery by phosphorylating and inactivating pro-apoptotic proteins, such as Bad. It also phosphorylates and activates IKKα, which, in turn, phosphorylates IκBα, triggering its degradation and leading to the nuclear translocation and activation of NF-κB and NF-κB-dependent pro-survival genes [54, 55]. Therefore, the apoptosis induction and inhibition of NF-κB signaling pathway caused by aspirin combined with cisplatin might be resulted from the inactivation of PI3K/Akt signaling. Of course, considering the results from Figure 2E and 2F that the inactivation of PI3K/AKT or ERK pathways combined with aspirin and cisplatin co-treatment induced more cell viability blockade compared to inactivation treatment alone, which implies the existence of some other pathways besides PI3K/AKT and ERK to be involved in the synergized anti-cancer effect mediated by Aspirin combined with Cisplatin, it is not excluded that the combined drug treatment directly activates pro-apoptotic signals and dysregulates NF-κB signaling independent of PI3K/Akt signaling, just as what was shown in the schematic in Figure 7. All these points deserve to be better explored in our further study.

Yamamoto et al. previously demonstrated that aspirin inhibits the activation of NF-κB pathway by inhibiting IKKβ activity in colon cancer cells and suggested that this specific interference may be involved in the antineoplastic property [56]. Another study by Li Ming et al. indicated that Aspirin irreversibly inhibited enzymatic activity of COX-2, a downstream oncogenic gene regulated by NF-κB, and blocked PGE_2_ production in esophageal cancer cells [57]. Consistently, we found that Aspirin combined with Cisplatin synergistically inhibited nuclear translocation of NF-κB p65/p50 and prevented p65/p50 binding to COX-2 promoter, finally suppressing COX-2 expression and PGE_2_ production. Of note, in line with the inactivation of PI3K/AKT or ERK signaling pathway, the inhibition of COX-2 activity does not necessarily extinguish cell viability suppression caused by Aspirin and Cisplatin co-treatment based on the data from Figure 5C, similarly suggesting the involvement of other mechanisms besides NF-κB/ COX-2 in the synergized anti-cancer effect mediated by Aspirin combined with Cisplatin, for instance, PI3K/AKT and ERK pathways. By referring to David’s review [58], we drew a schematic representation to elucidate the underlying molecular mechanisms involved in synergistic anti-tumor efficacy of this combined therapy on colon cancer (Figure 7).

In addition, the almost identical mechanisms of Aspirin sensitizing Cisplatin-mediated antineoplastic effect have been validated in our in vivo experiments. More importantly, although the clinical trial performed by Crabb et al. was not powered to test the hypothesis that Aspirin could reduce Cisplatin-induced ototoxicity [59], our results at least imply that in synergy with Aspirin, the equal inhibiting effect on colon carcinogenesis could be attained by applying lower-dose Cisplatin. In other words, more strikingly, we found that this combined therapy was capable to relieve the toxic side effects caused by using Cisplatin alone at high dosage, but to get the same or even better treatment outcome. Our results taken together suggest that Aspirin combined with Cisplatin has the potential to become a novel approach for colon cancer treatment.

## CONCLUSIONS

In summary, our study demonstrated that Aspirin sensitized the chemotherapeutic effect of Cisplatin in colon cancer as evidenced by the enhanced anti-proliferation, anti-migration, and proapoptotic activities in vitro and in vivo. Furthermore, we provided the mechanistic insights into such combinational treatment and found that the combined effects of Aspirin and Cisplatin might be achieved through better activating cytochrome-c-triggered apoptotic signaling, and more significantly inhibiting PI3K/AKT/ERK and NF-κB/COX-2 pathway. Our data therefore illustrated the potential of Aspirin in elevating Cisplatin’s anti-tumor effects in colon cancer, and also provided the possibility to develop the combinational use of Aspirin and Cisplatin as a novel therapeutic strategy for colon cancer treatment.

## MATERIALS AND METHODS

### Cell lines and cell culture

Human colon cancer cell lines SW620, LoVo, RKO and DLD-1 were obtained from ATCC (American Type Culture Collection). SW620 and RKO were cultured in DMEM medium (Invitrogen, Carlsbad, CA), meanwhile LoVo and DLD-1 cultured in RPMI 1640 medium (Invitrogen, Carlsbad, CA), supplemented with 10% fetal bovine serum (Gibco). All cells were cultured in the incubator (Thermo Fisher Scientific) with 5% CO_2_ at 37 °C consistently.

### Reagents and antibodies

Cisplatin and Aspirin applied to in vitro tests were purchased from Sigma-Aldrich. Antibodies against p50 (Cat: sc-8414), Bcl-2 (Cat: sc-7382) and Cytochrome C (Cat: sc-13560) were purchased from Santa Cruz Biotechnology. Antibodies against β-catenin (Cat: 8480T), Vimentin (Cat: 5741T), Snail (Cat: 3879T), Slug (Cat: 9585T), p-p65 (Cat: 3033S), p65 (Cat: 8242T), p110-α (Cat: 4255S), p110-β (Cat: 3011T), p110-γ (Cat: 5405T), p-PI3K p85(Tyr 458)/p55 (Tyr 199) (Cat: 4228S), p-PDK1 (Cat: 3438T), Akt (Cat: 9272S), p-Akt(Ser 473) (Cat: 4060T), Pten (Cat: 9188T), p-c-Raf (Cat: 9421T), p-mek1/2 (Cat: 9154T), Erk1/2 (Cat: 4695T), p-Erk1/2 (Cat: 4370T), COX-2 (Cat: 12282T), IKKα (Cat: 11930S), IKKβ (Cat: 8943S), p-IKKα/β (Cat: 2697T), IκB-α (Cat: 4814T) and p-IκB-α (Cat: 2859T) were purchased from Cell Signaling Technology. Antibodies against N-Cadherin (Cat: 22018-1-AP), β-actin (Cat: 20536-1-AP), MMP2 (Cat: 10373-2-AP), MMP9 (Cat: 10375-2-AP), Bax (Cat: 50599-2-Ig), PARP (Cat: 13371-1-AP), Lamin B1 (Cat: 12987-1-AP) and c-myc (Cat: 10828-1-AP) were purchased from Proteintech group. The NF-κB signaling inhibitor QNZ was purchased from Selleck Chemicals, China. Unless peculiarly noted, other chemicals were purchased from Sigma-Aldrich.

### siRNA sequences and transfection

The siRNAs targeting Akt, MEK1 and MEK2 were purchased from Shanghai GenePharma Company (Shanghai, China). The Akt siRNA sequence was 5'-CUCACAGCCCUGAAGUACUtt-3'. MEK1 siRNA sequence was 5'-AAGCAACTCATGGTTCATGCTTT-3'. MEK2 siRNA sequence was 5'-AAGAAGGAGAGCCTCACAGCA-3'. Cells plated into 96-well plates (5000 cells/well) were transfected with siRNA duplexes (1-2 μg) encapsulated by Lipofectamine 3000 (Invitrogen, Carlsbad, CA). At 48 hours after treatment, cell viabilities were tested by MTT analysis.

### Cell viability assay

About 5000 cells were seeded in each well of 96-well plates. After culture overnight, they were treated with different concentrations of Aspirin and/or chemotherapy drug Cisplatin for 48 h. Cell viability was measured by MTT assay. The drug concentration required to cause 50% cell growth inhibition (IC50) was determined by interpolation from dose-response curves.

### Colony formation assay

After treatment with Cisplatin alone or Aspirin alone or their combination for 48 hours, RKO and LoVo were harvested and seeded into six-well plates (2×10^3^ cells per well), and then they were cultured for 10 days. The cell clones were washed with PBS, and then fixed in methanol for 10 minutes, finally stained with 0.1% crystal violet. Colonies that contained more than 50 cells were counted.

### Wound-healing assay

Wound-healing assay was performed to detect cell migration ability. RKO and LoVo were seeded into six-well plates and then cultured to 90% confluence. Cell monolayers were wounded by a sterile 200μl pipette tip, and then washed with PBS for three times to remove detached cells. Cells were treated with indicated doses of Cisplatin alone or Aspirin alone or their combination for 56 hours. Then medium was replaced with PBS, the wound gap was observed and photographed by inverted microscope.

### Cell invasion assay

The transwell assay was performed to test the cellular capacity of invasion. RKO and LoVo were cultured in medium containing Cisplatin and/or Aspirin for 48 hours, then collected and about 10^4^ cells were resuspended in 100μl serum-free medium. The upper chamber was coated with Matrigel in 50μl serum-free medium and dried naturally. The lower chamber were filled with 500μl medium containing 20% FBS. After culture for 96 hours, invasive cells were stained with 0.1% crystal violet.

### Western blot analysis

Proteins from cell or tissue lysate were separated by 10% SDS-PAGE gel and electrophoretically transferred to PVDF membranes. After being blocked by 5% disposable milk for 2 hours at RT (room temperature), the protein bands were incubated with specific antibodies against β-actin (1:5000), Lamin B1 (1:2000), COX-2 (1:1000), or other antibodies from EMT kit, PI3K signaling pathway kit, ERK signaling pathway kit and NF-κB signaling pathway kit overnight at 4°C. After washing 3 times with TBST, the membranes were incubated with the second antibodies at RT for 2 hours. Finally the protein bands were detected by enhanced chemiluminescence according to the manufacturer's instructions.

### Acridine orange/ethidium bromide (AO/EB) fluorescence staining

RKO and LoVo plated in 6-well plates were treated with Cisplatin alone or Aspirin alone or their combination for 48 hours. Then 250μl AO/EB (10μg/ml) was gently added into each well. The apoptotic, necrotic and live cells were observed by a inverted fluorescence microscope (IX81, Olympus).

### Apoptosis assay

Drug-induced apoptosis was detected by FACS analysis using an Annexin V-FITC/PI staining kit (KeyGEN BioTECH, China). RKO and LoVo seeded into six-well plates were cultured overnight. After treatment with Cisplatin alone or Aspirin alone or their combination for 48 hours, cells were collected and washed once with cold PBS, subsequently stained with FITC-labeled Annexin V and PI. The stained cells were analyzed by flow cytometry (BD Accuri™ C6).

### Confocal immunofluorescence assay

Cells grown onto glass slides located into the 6-well plate were treated with Cisplatin and/or Aspirin for 48 hours. Then the samples were fixed with 4% paraformaldehyde for 30 min, permeabilized with PBST (PBS with 0.2% Triton-X100) for 5 min, and blocked with 10% BSA for 1 hour. Antibodies against Cytochrome-c, p65 and p50 diluted in PBS containing 1% BSA (1:200) were added to the cells and incubated overnight at 4°C. After washing with PBS, slides were incubated with fluorescein isothiocyanate- and rhodamine- conjugated secondary antibodies for 1 hour. The nuclei were counterstained with 4′,6-diamidino-2phenylindolde (DAPI, 5mg/ml storage solution diluted to 1:5000 with PBS). After five additional 10 minutes washes, samples were detected with Leica confocal microscope.

### Pulldown assay

The biotin-labeled double-stranded oligonucleotide probe, which corresponds to -892/+9 fragments of COX-2 promoter sequence were synthesized by PCR using biotin-labeled primers from TAKARA Company (sense, 5′-ACGTGACTTCCTCGACCCTC-3′; antisense, 5′-AA GACTGAAAACCAAGCCCA-3′). After treatment with Cisplatin and/or Aspirin for 48 hours, 400 μg nuclear protein, 4 μg DNA probe and 50 μl streptavidin-conjugated agarose beads were mixed and rotated gently at 4°C overnight. The beads complexed with binding proteins were obtained through centrifugation. After being washed with PBSI twice, the beads were resuspended by 2× loading buffer and boiled at 100°C for 10 min. Ultimately, the supernatant containing the pulled down proteins were detected by western blot using antibodies against p65 and p50.

### Dual-luciferase reporter assay

RKO and LoVo cells seeded in 6-well plates were transfected with COX-2 promoter (-892/+9 fragments) driven-luciferase plasmids and pRL-TK Renilla luciferase constructs (Promega) by Lipofectamine 3000 according to the manufacturer's instructions. After 24 hours, the cells were treated with Cisplatin and/or Aspirin for 48 hours. Then luciferase activities were measured according to the Dual-Luciferase Assay System protocol (Promega).

### PGE_2_ assay

RKO and LoVo plated in 6-well plates were treated with Cisplatin and/or Aspirin for 48 hours, then cell culture media were collected. The amounts of PGE_2_ in the media were detected by Prostaglandin E_2_ High Sensitivity in vitro competitive ELISA Kit (Abcam 133055).

### Mouse xenograft

All animal experiments were approved by the Animal Research Committee of Dalian Medical University and performed in accordance with established guidelines. LoVo cells (5×10^6^ or 5×10^5^) suspended in 100μl PBS were subcutaneously injected into the left or right flank of each male BALB/c nude mice aged 4-5 weeks (Charles River). Three days later, when the diameter of the tumor formed in the left flank by injecting 5×10^6^ LoVo cells reached 3mm×4mm, the mice were randomly separated into four groups (6 mice each group). The mice were treated with DMSO, Aspirin (100 mg/kg) by intragastric administration every day, and/or DDP (3 mg/kg) by intraperitoneal injection every three days according to the reported dosage application [18]. The tumor size was measured with a caliper once every three days and volumes were calculated as following: V=(width^2^×length)/2. On day 21 after tumor cell inoculation, all experimental mice were sacrificed, and the tumor xenografts were harvested and weighted. A portion of tumor tissues were fixed in 10% formalin and embedded in paraffin for immunohistochemical staining with antibodies against N-Cadherin (1:100), P-Akt (1:100), p-p65 (1:50), p65 (1:50), p50 (1:50), p-Erk1/2 (1:200), and COX-2 (1:50). Meanwhile, at the endpoint of therapy, blood samples of mice in each group were collected for renal toxicity testing. Serum levels of creatinine (Cr) and Blood Urea Nitrogen (BUN) were measured by detection kit (Cat: C011-2-1 and C013-2-1 from Nanjing Jiancheng Bioengineering Institute, China).

### Statistical analysis

The data were presented as the means ± standard deviation (SD) of at least three independent experiments. The error bars indicate the SD. Analysis of variance and Student’s t test were used to compare the values of the test and control samples. *, P<0.05 was considered to be significant; **, P<0.01 and ***, P<0.001 was considered to be strongly significant.

### Ethics approval

All animal maintenance and procedures were carried in accordance with the National Institute of Health Guide for the Care and Use of Laboratory Animals, with the approval of the Animal Research Committee of Dalian Medical University.

## References

[1] Brenner H, Kloor M, Pox CP. Colorectal cancer. Lancet. 2014; 383:1490–502. 10.1016/S0140-6736(13)61649-9 24225001

[2] Weitz J, Koch M, Debus J, Höhler T, Galle PR, Büchler MW. Colorectal cancer. Lancet. 2005; 365:153–65. 10.1016/S0140-6736(05)17706-X 15639298

[3] Yang B, Tang F, Zhang B, Zhao Y, Feng J, Rao Z. Matrix metalloproteinase-9 overexpression is closely related to poor prognosis in patients with colon cancer. World J Surg Oncol. 2014; 12:24. 10.1186/1477-7819-12-24 24476461 PMC3906768

[4] Tebbutt NC, Cattell E, Midgley R, Cunningham D, Kerr D. Systemic treatment of colorectal cancer. Eur J Cancer. 2002; 38:1000–15. 10.1016/S0959-8049(02)00062-X 11978525

[5] Desoize B, Madoulet C. Particular aspects of platinum compounds used at present in cancer treatment. Crit Rev Oncol Hematol. 2002; 42:317–25. 10.1016/S1040-8428(01)00219-0 12050023

[6] Galluzzi L, Senovilla L, Vitale I, Michels J, Martins I, Kepp O, Castedo M, Kroemer G. Molecular mechanisms of cisplatin resistance. Oncogene. 2012; 31:1869–83. 10.1038/onc.2011.384 21892204

[7] Siddik ZH. Cisplatin: mode of cytotoxic action and molecular basis of resistance. Oncogene. 2003; 22:7265–79. 10.1038/sj.onc.1206933 14576837

[8] Galluzzi L, Vitale I, Michels J, Brenner C, Szabadkai G, Harel-Bellan A, Castedo M, Kroemer G. Systems biology of cisplatin resistance: past, present and future. Cell Death Dis. 2014; 5:e1257. 10.1038/cddis.2013.428 24874729 PMC4047912

[9] Burn J, Sheth H. The role of aspirin in preventing colorectal cancer. Br Med Bull. 2016; 119:17–24. 10.1093/bmb/ldw028 27543497

[10] Coghill AE, Newcomb PA, Campbell PT, Burnett-Hartman AN, Adams SV, Poole EM, Potter JD, Ulrich CM. Prediagnostic non-steroidal anti-inflammatory drug use and survival after diagnosis of colorectal cancer. Gut. 2011; 60:491–98. 10.1136/gut.2010.221143 21051449 PMC3049822

[11] Drew DA, Chin SM, Gilpin KK, Parziale M, Pond E, Schuck MM, Stewart K, Flagg M, Rawlings CA, Backman V, Carolan PJ, Chung DC, Colizzo FP 3rd, et al. ASPirin Intervention for the REDuction of colorectal cancer risk (ASPIRED): a study protocol for a randomized controlled trial. Trials. 2017; 18:50. 10.1186/s13063-016-1744-z 28143522 PMC5286828

[12] Chan AT, Ogino S, Fuchs CS. Aspirin use and survival after diagnosis of colorectal cancer. JAMA. 2009; 302:649–58. 10.1001/jama.2009.1112 19671906 PMC2848289

[13] Herschman HR. Prostaglandin synthase 2. Biochim Biophys Acta. 1996; 1299:125–40. 10.1016/0005-2760(95)00194-8 8555245

[14] Vane JR. Inhibition of prostaglandin synthesis as a mechanism of action for aspirin-like drugs. Nat New Biol. 1971; 231:232–35. 10.1038/newbio231232a0 5284360

[15] Wang Y, Chen X, Zhu W, Zhang H, Hu S, Cong X. Growth inhibition of mesenchymal stem cells by aspirin: involvement of the WNT/beta-catenin signal pathway. Clin Exp Pharmacol Physiol. 2006; 33:696–701. 10.1111/j.1440-1681.2006.04432.x 16895542

[16] Chen J, Stark LA. Aspirin Prevention of Colorectal Cancer: Focus on NF-κB Signalling and the Nucleolus. Biomedicines. 2017; 5:E43. 10.3390/biomedicines5030043 28718829 PMC5618301

[17] Ban JO, Oh JH, Hwang BY, Moon DC, Jeong HS, Lee S, Kim S, Lee H, Kim KB, Han SB, Hong JT. Inflexinol inhibits colon cancer cell growth through inhibition of nuclear factor-kappaB activity via direct interaction with p50. Mol Cancer Ther. 2009; 8:1613–24. 10.1158/1535-7163.MCT-08-0694 19509257

[18] Liao D, Zhong L, Duan T, Zhang RH, Wang X, Wang G, Hu K, Lv X, Kang T. Aspirin Suppresses the Growth and Metastasis of Osteosarcoma through the NF-κB Pathway. Clin Cancer Res. 2015; 21:5349–59. 10.1158/1078-0432.CCR-15-0198 26202947

[19] Osaki M, Oshimura M, Ito H. PI3K-Akt pathway: its functions and alterations in human cancer. Apoptosis. 2004; 9:667–76. 10.1023/b:appt.0000045801.15585.dd 15505410

[20] Waseem T, Duxbury M, Ashley SW, Robinson MK. Ghrelin promotes intestinal epithelial cell proliferation through PI3K/Akt pathway and EGFR trans-activation both converging to ERK 1/2 phosphorylation. Peptides. 2014; 52:113–21. 10.1016/j.peptides.2013.11.021 24365237

[21] Lien GS, Lin CH, Yang YL, Wu MS, Chen BC. Ghrelin induces colon cancer cell proliferation through the GHS-R, Ras, PI3K, Akt, and mTOR signaling pathways. Eur J Pharmacol. 2016; 776:124–31. 10.1016/j.ejphar.2016.02.044 26879868

[22] Zhu L, Derijard B, Chakrabandhu K, Wang BS, Chen HZ, Hueber AO. Synergism of PI3K/Akt inhibition and Fas activation on colon cancer cell death. Cancer Lett. 2014; 354:355–64. 10.1016/j.canlet.2014.08.038 25199763

[23] Ong Q, Guo S, Zhang K, Cui B. U0126 protects cells against oxidative stress independent of its function as a MEK inhibitor. ACS Chem Neurosci. 2015; 6:130–37. 10.1021/cn500288n 25544156 PMC4304487

[24] Zhu QC, Gao RY, Wu W, Qin HL. Epithelial-mesenchymal transition and its role in the pathogenesis of colorectal cancer. Asian Pac J Cancer Prev. 2013; 14:2689–98. 10.7314/APJCP.2013.14.5.2689 23803016

[25] Zhu J, Liu C, Liu F, Wang Y, Zhu M. Knockdown of PFTAIRE Protein Kinase 1 (PFTK1) Inhibits Proliferation, Invasion, and EMT in Colon Cancer Cells. Oncol Res. 2016; 24:137–44. 10.3727/096504016X14611963142218 27458094 PMC7838739

[26] Baig S, Seevasant I, Mohamad J, Mukheem A, Huri HZ, Kamarul T. Potential of apoptotic pathway-targeted cancer therapeutic research: where do we stand? Cell Death Dis. 2016; 7:e2058. 10.1038/cddis.2015.275 26775709 PMC4816162

[27] Abraha AM, Ketema EB. Apoptotic pathways as a therapeutic target for colorectal cancer treatment. World J Gastrointest Oncol. 2016; 8:583–91. 10.4251/wjgo.v8.i8.583 27574550 PMC4980648

[28] Sakamoto K, Maeda S, Hikiba Y, Nakagawa H, Hayakawa Y, Shibata W, Yanai A, Ogura K, Omata M. Constitutive NF-kappaB activation in colorectal carcinoma plays a key role in angiogenesis, promoting tumor growth. Clin Cancer Res. 2009; 15:2248–58. 10.1158/1078-0432.CCR-08-1383 19276252

[29] Luo Y, Wang SX, Zhou ZQ, Wang Z, Zhang YG, Zhang Y, Zhao P. Apoptotic effect of genistein on human colon cancer cells via inhibiting the nuclear factor-kappa B (NF-κB) pathway. Tumour Biol. 2014; 35:11483–88. 10.1007/s13277-014-2487-7 25128065

[30] Stark LA, Din FV, Zwacka RM, Dunlop MG. Aspirin-induced activation of the NF-kappaB signaling pathway: a novel mechanism for aspirin-mediated apoptosis in colon cancer cells. FASEB J. 2001; 15:1273–75. 10.1096/fj.00-0529fje 11344111

[31] Pålsson-McDermott EM, O’Neill LA. Signal transduction by the lipopolysaccharide receptor, Toll-like receptor-4. Immunology. 2004; 113:153–62. 10.1111/j.1365-2567.2004.01976.x 15379975 PMC1782563

[32] Yoon YK, Woo HJ, Kim Y. Orostachys japonicus Inhibits Expression of the TLR4, NOD2, iNOS, and COX-2 Genes in LPS-Stimulated Human PMA-Differentiated THP-1 Cells by Inhibiting NF-κB and MAPK Activation. Evid Based Complement Alternat Med. 2015; 2015:682019. 10.1155/2015/682019 25810745 PMC4355124

[33] Maldonado ME, Bousserouel S, Gossé F, Lobstein A, Raul F. Implication of NF-κB and p53 in the expression of TRAIL-death receptors and apoptosis by apple procyanidins in human metastatic SW620 cells. Biomedica. 2010; 30:577–86. 10.7705/biomedica.v30i4.296 21713362

[34] Buhrmann C, Mobasheri A, Busch F, Aldinger C, Stahlmann R, Montaseri A, Shakibaei M. Curcumin modulates nuclear factor kappaB (NF-kappaB)-mediated inflammation in human tenocytes in vitro: role of the phosphatidylinositol 3-kinase/Akt pathway. J Biol Chem. 2011; 286:28556–66. 10.1074/jbc.M111.256180 21669872 PMC3151097

[35] Jakobsson PJ, Thorén S, Morgenstern R, Samuelsson B. Identification of human prostaglandin E synthase: a microsomal, glutathione-dependent, inducible enzyme, constituting a potential novel drug target. Proc Natl Acad Sci USA. 1999; 96:7220–25. 10.1073/pnas.96.13.7220 10377395 PMC22058

[36] Marnett LJ, DuBois RN. COX-2: a target for colon cancer prevention. Annu Rev Pharmacol Toxicol. 2002; 42:55–80. 10.1146/annurev.pharmtox.42.082301.164620 11807164

[37] Gallouet AS, Travert M, Bresson-Bepoldin L, Guilloton F, Pangault C, Caulet-Maugendre S, Lamy T, Tarte K, Guillaudeux T. COX-2-independent effects of celecoxib sensitize lymphoma B cells to TRAIL-mediated apoptosis. Clin Cancer Res. 2014; 20:2663–73. 10.1158/1078-0432.CCR-13-2305 24637636

[38] Goel A, Boland CR. Epigenetics of colorectal cancer. Gastroenterology. 2012; 143:1442–1460.e1. 10.1053/j.gastro.2012.09.032 23000599 PMC3611241

[39] André T, Boni C, Mounedji-Boudiaf L, Navarro M, Tabernero J, Hickish T, Topham C, Zaninelli M, Clingan P, Bridgewater J, Tabah-Fisch I, de Gramont A, and Multicenter International Study of Oxaliplatin/5-Fluorouracil/Leucovorin in the Adjuvant Treatment of Colon Cancer (MOSAIC) Investigators. Oxaliplatin, fluorouracil, and leucovorin as adjuvant treatment for colon cancer. N Engl J Med. 2004; 350:2343–51. 10.1056/NEJMoa032709 15175436

[40] Bang SM, Heo DS, Lee KH, Byun JH, Chang HM, Noh DY, Choe KJ, Bang YJ, Kim SR, Kim NK. Adjuvant doxorubicin and cyclophosphamide versus cyclophosphamide, methotrexate, and 5-fluorouracil chemotherapy in premenopausal women with axillary lymph node positive breast carcinoma. Cancer. 2000; 89:2521–26. 10.1002/1097-0142(20001215)89:12<2521::AID-CNCR2>3.0.CO;2-F 11135211

[41] Son DJ, Hong JE, Ban JO, Park JH, Lee HL, Gu SM, Hwang JY, Jung MH, Lee DW, Han SB, Hong JT. Synergistic Inhibitory Effects of Cetuximab and Cisplatin on Human Colon Cancer Cell Growth via Inhibition of the ERK-Dependent EGF Receptor Signaling Pathway. Biomed Res Int. 2015; 2015:397563. 10.1155/2015/397563 26491668 PMC4600871

[42] Macciò A, Madeddu C. Cisplatin : an old drug with a newfound efficacy — from mechanisms of action to cytotoxicity. Expert Opin Pharmacother. 2013; 14:1839–57. 10.1517/14656566.2013.813934 23876094

[43] Bibbins-Domingo K, Force US, and U.S. Preventive Services Task Force. Aspirin Use for the Primary Prevention of Cardiovascular Disease and Colorectal Cancer: U.S. Preventive Services Task Force Recommendation Statement. Ann Intern Med. 2016; 164:836–45. 10.7326/M16-0577 27064677

[44] Chan AT, Giovannucci EL, Schernhammer ES, Colditz GA, Hunter DJ, Willett WC, Fuchs CS. A prospective study of aspirin use and the risk for colorectal adenoma. Ann Intern Med. 2004; 140:157–66. 10.7326/0003-4819-140-3-200402030-00006 14757613

[45] Bradley MC, Black A, Freedman AN, Barron TI. Prediagnostic aspirin use and mortality in women with stage I to III breast cancer: A cohort study in the Prostate, Lung, Colorectal, and Ovarian Cancer Screening Trial. Cancer. 2016; 122:2067–75. 10.1002/cncr.30004 27149646

[46] Luciani MG, Campregher C, Gasche C. Aspirin blocks proliferation in colon cells by inducing a G1 arrest and apoptosis through activation of the checkpoint kinase ATM. Carcinogenesis. 2007; 28:2207–17. 10.1093/carcin/bgm101 17510082

[47] Yu HG, Huang JA, Yang YN, Huang H, Luo HS, Yu JP, Meier JJ, Schrader H, Bastian A, Schmidt WE, Schmitz F. The effects of acetylsalicylic acid on proliferation, apoptosis, and invasion of cyclooxygenase-2 negative colon cancer cells. Eur J Clin Invest. 2002; 32:838–46. 10.1046/j.1365-2362.2002.01080.x 12423325

[48] Dong H, Liu G, Jiang B, Guo J, Tao G, Yiu W, Zhou J, Li G. The effects of aspirin plus cisplatin on SGC7901/CDDP cells *in vitro.* Biomed Rep. 2014; 2:344–48. 10.3892/br.2014.241 24748972 PMC3990199

[49] Kilari RS, Bashir AIJ, Devitt A, Perry CJ, Safrany ST, Nicholl ID. The Cytotoxicity and Synergistic Potential of Aspirin and Aspirin Analogues Towards Oesophageal and Colorectal Cancer. Curr Clin Pharmacol. 2019; 14:141–151. 10.2174/1574884713666181112141151 30417794 PMC7040498

[50] Cheng Q, Shi H, Wang H, Min Y, Wang J, Liu Y. The ligation of aspirin to cisplatin demonstrates significant synergistic effects on tumor cells. Chem Commun (Camb). 2014; 50:7427–30. 10.1039/C4CC00419A 24777030

[51] Bastiaannet E, Sampieri K, Dekkers OM, de Craen AJ, van Herk-Sukel MP, Lemmens V, van den Broek CB, Coebergh JW, Herings RM, van de Velde CJ, Fodde R, Liefers GJ. Use of aspirin postdiagnosis improves survival for colon cancer patients. Br J Cancer. 2012; 106:1564–70. 10.1038/bjc.2012.101 22454078 PMC3341868

[52] Cross DA, Alessi DR, Cohen P, Andjelkovich M, Hemmings BA. Inhibition of glycogen synthase kinase-3 by insulin mediated by protein kinase B. Nature. 1995; 378:785–89. 10.1038/378785a0 8524413

[53] Lawlor MA, Alessi DR. PKB/Akt: a key mediator of cell proliferation, survival and insulin responses? J Cell Sci. 2001; 114:2903–10. 11686294 10.1242/jcs.114.16.2903

[54] Ozes ON, Mayo LD, Gustin JA, Pfeffer SR, Pfeffer LM, Donner DB. NF-kappaB activation by tumour necrosis factor requires the Akt serine-threonine kinase. Nature. 1999; 401:82–85. 10.1038/43466 10485710

[55] Barkett M, Gilmore TD. Control of apoptosis by Rel/NF-kappaB transcription factors. Oncogene. 1999; 18:6910–24. 10.1038/sj.onc.1203238 10602466

[56] Yamamoto Y, Yin MJ, Lin KM, Gaynor RB. Sulindac inhibits activation of the NF-kappaB pathway. J Biol Chem. 1999; 274:27307–14. 10.1074/jbc.274.38.27307 10480951

[57] Li M, Lotan R, Levin B, Tahara E, Lippman SM, Xu XC. Aspirin induction of apoptosis in esophageal cancer: a potential for chemoprevention. Cancer Epidemiol Biomarkers Prev. 2000; 9:545–49. 10868686

[58] Tougeron D, Sha D, Manthravadi S, Sinicrope FA. Aspirin and colorectal cancer: back to the future. Clin Cancer Res. 2014; 20:1087–94. 10.1158/1078-0432.CCR-13-2563 24327271 PMC3947439

[59] Crabb SJ, Martin K, Abab J, Ratcliffe I, Thornton R, Lineton B, Ellis M, Moody R, Stanton L, Galanopoulou A, Maishman T, Geldart T, Bayne M, et al. COAST (Cisplatin ototoxicity attenuated by aspirin trial): A phase II double-blind, randomised controlled trial to establish if aspirin reduces cisplatin induced hearing-loss. Eur J Cancer. 2017; 87:75–83. 10.1016/j.ejca.2017.09.033 29128692 PMC5729023

